# Perception and attitude of Korean physicians towards generic drugs

**DOI:** 10.1186/s12913-017-2555-y

**Published:** 2017-08-29

**Authors:** Mikyung Ryu, Juyoung Kim

**Affiliations:** Pharmaceutical Benefits, Management division, Health Insurance Review & Assessment Service, 304 Hyoryeong-ro, Seocho-gu, Seoul, 06720 South Korea

**Keywords:** South Korea, Physician, New drug policy, Generic drugs

## Abstract

**Background:**

In 2012, a new pharmaceutical policy was introduced in Korea. According to the new policy, off-patent brand-name drugs (original drugs) and generic drugs must be priced the same.

**Methods:**

This study aims to investigate the perception and attitude of Korean physicians towards generic drugs before and after the policy reform. Surveys were conducted with registered doctors at the Health Insurance Review Agency (HIRA) twice, in 2011 and 2013, by means of email and HIRA online survey systems.

**Results:**

In the 2011 survey, 82% knew about the bioequivalent (BE) guideline, whereas only 25.7% trusted BE testing results. More than half preferred original drugs to generic drugs because of clinical experience and generic drugs confidence limits. 64.2% pointed out that the Korean generic drugs prices are more expensive than in other counties. In the 2013 survey, 73% preferred original drugs to generic drugs because of believed difference in drug effectiveness. After the pricing policy reform, 35.5% stated that they didn’t change their prescribing pattern, whereas 29.7% stated that they began prescribing generic drugs.

**Conclusions:**

The Korean government has revised and strengthen the guideline on BE test to improve the quality and confidence of generic drugs. Although generic drugs prescription was increased slightly more than the 2011 survey, 2013 survey showed that around 70% of respondents still preferred original drugs.

## Background

In 2013, health spending (excluding investment expenditure in the health sector) accounted for 6.9% of the GDP in Korea, still well below the average of 8.9% in OECD (Organization for Economic Co-operation and Development) countries [1]. However, between 2008 and 2013**,** growth in health expenditures was 1.1%, which is higher than the OECD average of 0.6% [2]. Particularly, pharmaceutical spending has historically undergone strong growth and has been one of the main contributing factors in the overall increase in health spending. As of 2013, the pharmaceutical expenditure rate of Korea was 20.6% of the total health expenditure higher than the OECD average of 16.6%, and ranked 7th among the 34 countries [1]. Thus, a major concern of the Korean government is controlling pharmaceutical expenditures. The Korean government adopted several pharmaceutical policies aiming to limit increases in pharmaceutical spending and facilitate cost-effective medication use.

Generic drugs are typically much less expensive than brand-name drug (hereafter referred to as “original drug”) equivalents [3]. The term “generic” has the same meaning as used by the FDA and EMEA [4–7]. Generic drugs can be deemed interchangeable without any concerns about safety and efficacy, as they are therapeutically equivalent to their original drug counterparts. Therefore, generic drugs utilization is an important strategy to reduce pharmaceutical expenditures. According to the Ministry of Food and Drug Safety (MFDS), generic drugs are identical or bioequivalent (BE) to original drugs in dosage form, safety, strength, route of administration, quality, performance characteristics and intended use [3]. Since 2000, all new pharmaceutically equivalent or alternative generic drugs have been required to prove therapeutic equivalence either by BE studies or comparative clinical trials [8]. In 2007, the MFDS revised the guideline on BE testing, amended the pharmaceutical affairs act and broadened the evaluation from single substances to combinations (KFDA notification 2007–65) [9]. Recently, the Korean government introduced a new pricing system starting in January 2012 requiring that BE drugs be priced the same (Ministry of Health and Welfare notification 2011-176) [10–12]. In the first year after patent expiration, the price of original drugs and generic drugs will be set at 70% and 59.5% of the original drug price respectively. Beginning in the second year after patent expiration, the price for all drugs will be set at 53.5% of the original drug price, regardless of the order of entry, eliminating differences between the prices of original drugs and generic drugs, as well as the difference in co-payments [13–15]. These rules were applied to 13,184 listed drugs, reducing the prices of 6504 drugs [14]. As a result, the average list price of listed drugs decreased by 14.2% [14].

In Korea, physicians can prescribe only by a specific brand name, not by the international non-proprietary name [14]. The cheaper, BE generic substitution is permitted, but when a substitution occurs, pharmacists must notify the prescribing physician, which is complicated and inconvenient. Because the physician may not agree with the pharmacist, the substitution rate is very low. Therefore, the physicians’ opinions on drug policies can be a key factor to increase the use of rational and cost-effective drugs and play a pivotal role in implementing the different pharmaceutical policies [16]. Few studies have been performed in Korea to investigate physicians’ understanding of generic drugs and to evaluate their attitudes toward generic drugs prescription before and after the pricing policy reform [13–15, 17, 18]. Our surveys were conducted with registered doctors at the government-affiliated Health Insurance Review Agency (HIRA) in 2011 and 2013. The 2011 primary survey provided baseline data to support the revision of pharmaceutical policy, especially drug pricing policy, by evaluating the physicians’ perception of generic drugs, reasonable pricing and current drug policy [19]. The 2013 survey investigated the physicians’ preference, acceptance and attitudes toward generic drugs and their prescribing patterns before and after the drug pricing policy reform in 2012.

## Methods

The sample group, which was obtained from the HIRA, was comprised of a list of 907 doctors in Korea. The respondents received and responded to the survey questionnaires through an email and the HIRA online survey systems. Several follow-up reminder email were used to increase the response rate. This study does not contain clinical data or patient data. The study setting and the complete anonymity of the respondents were in accordance with the national ethical instructions for researches [20]. Each doctor participated voluntarily. The informed consent of the participants was obtained and no personal data of the participants were reported. The 11 questions in the first survey focused on their perception of the BE guidelines, their confidence in and preference for generic drugs, their opinions on reasonable generic drugs’ price and their willingness to prescribe generic drugs. The 15 questions in the second survey were created to investigate the change of respondents’ preferences and attitudes towards generic drugs, the factors behind these choices and their opinions on the 2012 drug pricing policy. The first survey data was collected from June 7, 2011 to June 22, 2011. Three hundred sixty one doctors responded to the survey (response rate of 39.8%). Data was collected from 296 doctors who responded to the second survey (response rate of 32.6%) from Aug, 26, 2013 to Oct, 2, 2013. Data analysis was performed using SAS 8.2. Frequency and cross-tabulation were used for descriptive analysis. Chi-square statistic program was used to investigate the differences in the doctors’ responses between the different groups of doctors (sex, age, position, specialty, classification, provinces). A significance level of less than 0.05 was used. The odds ratios were calculated for the variables which demonstrated the significant differences based on the chi-square analysis.

## Results

### Respondent characteristics

The demographic characteristics of the survey participants are presented in Table 1 and Table 4.

**Table 1:** Characteristics of the respondents

Characteristic	Study 2011 *N* (%)	Study 2013 *N* (%)
All	361 (100.0)	296 (100.0)
Gender		
Male	329 (91.1)	275 (92.9)
Female	32 (8.9)	21 (7.1)
Age		
30-39 years	7 (1.9)	1 (0.3)
40-49 years	152 (42.1)	89 (30.1)
50-59 years	169 (46.8)	160 (54.1)
60 + years	33 (9.2)	46 (15.5)
Specialty		
Internal medicine	70 (19.4)	48 (16.2)
Surgery	129 (35.7)	79 (26.7)
Psychiatry and Neurology	27 (7.5)	16 (5.4)
Obstetrics and Gynecology, Pediatrics	48 (13.3)	35 (11.8)
Dermatology, Urology	29 (8.0)	25 (8.5)
Ophthalmology, Otolaryngology	33 (9.1)	43 (14.5)
Others	25 (7.0)	50 (16.9)
Type of clinical facility		
University hospital	206 (57.0)	149 (50.3)
General hospital	72 (20.0)	48 (16.2)
Hospital	17(4.7)	13 (4.4)
Clinic	59 (16.0)	77 (26.0)
Dental hospital	7(1.9)	9 (3.1)
Hospital location		
Seoul	115 (31.9)	96 (32.4)
Incheon/ Gyeonggi	84 (23.3)	66 (22.3)
Busan/ Ulsan/ Gyeong nam	50 (13.8)	35 (11.8)
Daegu/ Gyeong buk	31 (8.6)	28 (9.5)
Daejeon/ Chung cheong	35 (9.7)	34 (11.5)
GwangJu/ Jeonra	41 (11.4)	34 (11.5)
Gangwon	1 (0.3)	1 (0.3)
Jeju	4 (1.1)	2 (0.7)

### Knowledge of the regulatory BE guideline for generic drugs, the reliability of BE result and the perception of generic drugs

In the 2011 survey, when asked about their knowledge of the BE guidelines for the approval of generic drugs by the MFDS, the majority of the respondents (82.0%) selected “knew somewhat” or “knew well” about the BE guidelines. But only a total of 25.7% (*n* = 93) of the respondents felt that the MFDS’s BE testing results were reliable (Table 2). In addition, when the doctors were asked whether bio-equivalent generic drugs were therapeutically equivalent to original drugs, over half of the respondents (*n* = 192, 53.2%) believed there was a difference in the safety and effectiveness between generic drugs and original drugs, whereas 17.5% stated that generic drugs were therapeutically equivalent to original drug and the rest remained neutral (Table 2). The main reason for the lack of confidence in generic drugs was physicians’ clinical experience (32%) and the second reason was the lack of confidence in the BE testing results (26%). Also, generic drugs confidence limits, the evidence limits, the limit of effect influenced the lack of confidence in generic drugs (Table 2). Compared with the 2011 survey, the 2013 survey showed that the proportion of negative perception of generic drugs increased by nearly 20%; more than two-thirds (*n* = 210, 71%) had doubts about the safety and efficacy of generic drugs and only 15.3% believed that there was no difference in the safety and effectiveness between generic drugs and original drugs (Table 3). The results from this study were in line with the other surveys which were conducted by the Korean Medical Association newspaper in 2011 and 2015, [14, 15] which found that less than one-third of respondents (2011:30.3%; 2015:25%) believed that generic drugs were therapeutically equivalent to original drugs, or even to each other. Also, interestingly, almost all respondents in 2013 (93.6%) believed that there was difference in the safety and effectiveness between two generic drugs and only 6.4% of the respondents agreed with that some generic drugs were therapeutically equivalent to other generic drugs, to each other.

**Table 2:** Knowledge, perception and opinions towards the generic (2011)

Variables		Answer	*N*	%
Knowledge	BE criteria^a^	Not informed at all	12	3.3
		A little informed	53	14.7
		Moderately informed	228	63.2
		Completely informed	68	18.8
Reliability	BE results	Not at all confident	3	0.8
		Not very confident	79	21.9
		Neutral	186	51.6
		Very confident	90	24.9
		Completely confident	3	0.9
Perceptions	Safety and effectiveness compared to original	Non-equivalent	192	53.2
		Neutral	106	29.3
		Equivalent	63	17.5
	Main reasons for the negative recognition	Clinical experience	116	32
		Confidence limit in BE test	94	26
		Confidence limit in generic	94	26
		Effect limit	32	9
		Evidence limit	11	3
		Others (minor opinion)*	14	4
	Price	Low price	20	5.5
		Accurate price	109	30.2
		Over price	232	64.3
Opinions	Reasonable price of generic	Same price as original	11	3
		80% of original	93	25.8
		70% of original	95	26.3
		60% of original	85	23.5
		Less than 50% of original	77	21.3
	Factors associated with the generic substitution (subjective response)	Same equivalent effects and safety as the original	316	87.5
		Confidence of the pharmaceutical company	34	9.4
		Drug price	8	2.2
		Pharmaceutical marketing activity	2	0.6
		Hospital policy	1	0.3
	Factors to improve the generic prescription (multiple response ^b^)	Quality maintenance of generic	261	32.6
		BE confidence recovery	216	26.9
		Disclosure of the BE results	193	24
		Price cut	88	10.9
		Incentive policy	41	5
		Others	5	0.6
Prescription behavior	If generic quality would be improved	Switch to the generic	305	84.4
		No change	53	14.7
		Switch to the original	3	0.9

The BE test states that two treatments are not different from one another if the 90% confidence interval of the ratio of a log-transformed exposure measure (AUC and/or C_max_) falls completely within the range 80-125%

^a^BE criteria in south Korea MFDA

^b^Respondents could choose several options

**Table 3:** Perception, prescription behavior and opinions towards the generic (2013)

Category	Variables	Answer	%	*N*
Perceptions	Safe and effective compared to original	Non-equivalent	71	210
		Neutral	15.5	46
		equivalent	13.5	40
Prescription behavior	Patients’ OOP	Consider	87.2	258
		No consider	12.8	38
	After policy reform	Switch to the original	16.6	49
		Switch to the generic	29.7	88
		No switch	35.5	105
		Don’t know	18.2	54
	If the patients’ OOP burden is reduced	Switch to the generic	65.5	194
		No change	34.5	102
	If the incentive is offered	Switch to the generic	40.2	119
		No change	59.8	177
Opinions	Factors associated with generic substitution (subjective response)	Same equivalent effects and safety as the original	86.5	256
		Confidence of the pharmaceutical company	6.1	18
		Physicians’ experience	5.7	17
		Drug price	1.4	4
		Hospital policy	0.3	1
	Cost effective drug-use policy (subjective response)	Incentive policy	14.9	44
		Improvement of GD quality	12.8	38
		Offering of drug information	12.5	37
		Reasonable price	9.8	29
		Independent prescription rights	4.1	12
		Others^a^ (some diverse opinions)	46	136

^a^For example, manage the rebate and bribe from pharmaceutical company, targeted therapies, new drug development and control duplicate and inappropriate drug therapies

### Appropriate generic drugs’ price

In the 2011 survey, an inquiry concerning the generic drugs’ price**,** a total of 64.3% of respondents answered that generic drugs are expensive in Korea and almost all respondents (97%) answered that the appropriate generic drugs’ price was lower than the current price. Also, a total of 21.3% of respondents answered that the appropriate generic drugs’ price is lower than 50% of the original drugs’ price (Table 2). This results provided the baseline data to support a new national pharmaceutical pricing policy that the drugs composed of the same ingredients should have the same price starting in January 2012 [19].

### Preference between original drugs versus generic drugs

Concerning their preference between original drugs and generic drugs, 76.7% of the respondents overwhelmingly preferred original drugs, while 21.1% did not discriminate between original drugs and generic drugs, and only 2.2% preferred generic drugs (Table 4). A sub-group analysis was performed based on the doctors’ institute, medical specialty and hospital location, with a total of 83.5% of respondents who work at a University hospital preferring original drugs, whereas 57.6% of respondents who worked at a clinic preferred original drugs. By specialty, most respondents preferred original drugs, as follows: Dermatology/Urology (86.2%), Ophthalmology/Otolaryngology (81.8%), Obstetrics and Gynecology/Pediatrics (81.3%), Internal medicine (78.6%), Psychiatry/Neurology (77.8%), Surgery (74.4%), and Others (64.3%). By region, more than 70% of respondents in all provinces (excluding Jeju province) preferred original drugs. These results demonstrate that statistically significant difference might occur among doctors with regard to medicine preference (*p* = 0.0002). The 2013 survey showed that a total of 73.0% preferred original drugs and 15.5% did not discriminate between original drugs and generic drugs, while only 11.5% preferred generic drugs (Table 4). Compared with the 2011 survey, similar proportions of respondents stated that they preferred original drugs, with a preference for generic drugs increasing by 10%. A total of 83.8% of respondents who work at the university hospital preferred original drugs, whereas a total of 50.6% of respondents who work at the clinic preferred original drugs. By specialty, the majority of respondents preferred original drugs; Internal medicine (85.4%), Ophthalmology/Otolaryngology (79.1%), Psychiatry/Neurology (77.8%), Dermatology/Urology (72%), Obstetrics and Gynecology/Pediatrics (71.4%), Other (66%), and Surgery (63.3%). By region, more than 70% of respondents in Seoul, Inchon/Gyeonggi, Daegu/Gyoungbuk and Daejion/Chungcheong and less than 70% of respondents in Busan/Ulsan/Gyeognam, Gwangju/Geonra and Gangwon provinces stated that they preferred original drugs (76%, 74.2%, 78.6%, 76.5%, 65.7%, 61.8% and 0% respectively).

**Table 4:** Comparison of respondents preference by socio- demographic characteristic (2011 vs 21,013)

	2011 *N* (%)					2013 *N* (%)				
Variables	Total	Original	GD	Neutral	*p* values	Total	Original	GD	Neutral	*p* values
Total	361 (100.0)	277 (76.7%)	8 (2.2%)	76 (21.1%)		296 (100.0)	216 (73.0%)	34 (11.5%)	46 (15.5%)	
Type of clinical facility										
University hospital	206	172 (83.5%)	1 (0.5%)	33 (16.0%)	**	149	125 (83.8%)	8 (5.4%)	16 (10.8%)	**
General hospital	72	54 (75.0%)	3 (4.2%)	15 (20.8%)		48	40 (83.3%)	2 (4.2%)	6 (12.5%)	
Hospital	17	13 (76.5%)	0 (0%)	4 (23.5%)		13	9 (69.2%)	2 (15.4%)	2 (15.4%)	
Clinic	59	34 (57.6%)	3 (5.1%)	22 (37.3%)		77	39 (50.6%)	18 (23.4%)	2 (26.0%)	
Dental hospital	7	4 (57.1%)	1 (14.3%)	2 (28.6%)		9	3 (33.3%)	4 (44.4%)	2 (22.2%)	
Specialty										
Internal medicine	70	55 (78.6%)	2 (2.9%)	13 (18.6%)		48	41 (85.4%)	1 (2.1%)	6 (12.5%)	
Surgery	129	96 (74.4%)	2 (1.6%)	31 (24.0%)		79	50 (63.3%)	11 (14.0%)	18 (22.8%)	
Psychiatry and Neurology	27	21 (77.8%)	1 (3.7%)	5 (18.5%)		16	11 (68.8%)	3 (18.8%)	2 (12.5%)	
Obstetrics and Gynecology, Pediatrics	48	39 (81.3%)	0 (0%)	9 (18.8%)		35	25 (71.4%)	1 (2.9%)	9 (25.7%)	
Dermatology, Urology	29	25 (86.2%)	0 (0%)	4 (13.8%)		25	18 (72.0%)	2 (8.0%)	5 (20.0%)	
Ophthalmology, Otolaryngology	33	27 (81.8%)	1 (3.0%)	5 (15.2%)		43	34 (79.1%)	5 (11.6%)	4 (9.3%)	
Other	25	14 (56.0%)	2 (8.0%)	9 (36.0%)		50	33 (66.0%)	10 (20.0%)	7 (14.0%)	
Hospital location										
Seoul	115	90 (78.3%)	0 (0%)	25 (21.7%)	*	96	73 (76.0%)	9 (9.4%)	14 (14.6%)	*
Incheon/ Gyeonggi	84	65 (77.4%)	2 (2.4%)	17 (20.2%)		66	49 (74.2%)	5 (7.6%)	12 (18.2%)	
Busan/ Ulsan/ Gyeongnam	50	41 (82.0%)	0 (0%)	9 (18.0%)		35	23 (65.7%)	4 (11.4%)	8 (22.9%)	
Daegu/ Gyeongbuk	31	24 (77.4%)	1 (3.2%)	6 (19.4%)		28	22 (78.6%)	1 (3.6%)	5 (17.9%)	
Daejeon/ Chungcheong	35	25 (71.4%)	1 (2.9%)	9 (25.7%)		34	26 (76.5%)	4 (11.8%)	4 (11.8%)	
Gwangju/ Jeonra	41	30 (73.2%)	2 (4.9%)	9 (22.0%)		34	21 (61.8%)	10 (29.4%)	3 (8.8%)	
Gangwon	1	0 (0%)	0 (0%)	1 (100%)		1	0 (0%)	1 (100%)	0 (0%)	
Jeju	4	2 (50.0%)	2 (50.0%)	0 (0%)		2	2 (100%)	0 (0%)	0 (0%)	

*p<0.05, **p<0.01 using chi-square test

### Opinions on generic drugs prescriptions

When asked about the factors associated with a willingness to prescribe generic drugs, almost all respondents (87.5%) cited a belief that generic drugs are therapeutically equivalent and just as safe as originals. It was followed by confidence in the pharmaceutical company (9.4%), the price of drug (2.2%), pharmaceutical marketing activity (0.6%) and hospital policy (0.3%) (Table 2). Next, we studied the factors that would improve generic drugs prescription rates. The post marketing surveillance (PMS) system for quality maintenance (32.6%), confidence in the BE test (26.9%), and reliable BE results (24%) were the three most important factors that influenced the frequency of generic drugs prescription. Also price cuts (10.9%) and incentive policies for generic drugs prescribing (5%) were associated with increases in generic drugs prescription. 83% of respondents stated that they would be willing to prescribe the generic drugs more often, if the quality of generic drugs was improved by transparent, strong and efficient regulatory administration such as the mandatory installation of audit trail and good manufacturing practices (GMP), drug manufacturing form (DMF) enlargement and PMS, In the 2013 survey, when the physicians generally selected the medicine, almost all respondents (87.2%) stated that they considered the patient’s out-of-pocket (OOP) cost. If the patient’s OOP burden was reduced, 65.5% of respondents (*n* = 194) stated that they would be willing to switch to the less expensive generic drugs. If financial incentives were offered to physicians, 40.2% of respondents stated that they would be willing to switch to generic drugs, whereas 59.8% of respondents stated that they would continue prescribing original drugs. The primary factors affecting physicians’ decision to prescribe generic drugs, equivalent therapeutic effects and safety, remained the same in the 2011 survey (86.5%). Also, the trust of the pharmaceutical company (6.1%), physicians’ own experiences (5.7%), drug price (1.4%), and hospital policies (0.3%) were associated with generic drugs prescriptions. In the questions related to prescribing behavior after the pharmaceutical policy reform, 35.5% stated that they didn’t change their prescribing pattern, 29.7% stated that they switched to the generic drugs prescription, 16.6% stated that they switched to original drug prescription, while 18.2% said they did not know. Based on these findings, more than a third of the respondents did not change their prescription pattern, whereas about half of the respondents changed their prescribing preferences.

### Opinions on drug policies

In the 2013 study, the participating physicians’ opinions on the pharmaceutical policies towards cost- effective and rational use of drugs were shown in Table 4. The incentive policy introduction (14.9%), improvement of generic drugs quality (12.8%), and availability of drug information (12.5%) were the three major opinions for the improvement cost-effective drug prescription rate. There were followed by reasonable drug pricing (9.8%) and independent prescription rights (4.1%) and some minority opinions.

## Discussion

When generic drugs are approved in Korea, it has met the rigorous standards, guidelines and tests established by the MFDS with respect to the identity, strength, quality, purity, and potency [3, 6–9]. But, since the 2006 BE fabrication scandal, confidence was lost in generic drugs, particularly by physicians who prefer to prescribe original drugs [21, 22]. In 2006, the MFDS surveyed BE testing organizations and inspected 4285 items which were approved in the BE test. 18 test institutions, such as Lab Frontier, were found in suspicion of data manipulation, and the approvals were cancelled on 307 items of the 104 companies from May 30, 2006 to August 20, 2008 [23–25]. After this discovery, the Korean government reformed regulations concerning the sanction including the revocation [9, 26]. The MFDS has sought to strengthen the BE evaluation process and determine the current implementation status. But these survey results still showed poor confidence in the BE testing results certified by the MFDS (2011:25.7%; 2013:29%). Consequently, there was no change in the negative perception of the BE test results for generic drugs.

Regarding drug preferences, both studies showed that similar proportions of respondents preferred original drug (2011:76.7%; 2013:73%) and only a minority preferred generic drugs (2011: 2.2%; 2013:11.5%). In the sub-group analysis based on the type of clinical facility, the hospital location and the physicians’ medical specialty, almost all respondents (2011:83.5%; 2013:83.8%) who worked at university hospitals preferred original drugs, whereas half of the respondents who worked at clinics preferred original drugs (2011:57.6%; 2013:50.6%). The preference was different in several geographic areas. Our results showed that almost all the physicians in Korea who participated in this survey indicated a low generic drugs preference rate. This phenomenon was observed in all of the provinces and specialties. But the level of preference for original drugs was slightly different by healthcare institution, province and specialty; however, statistically significant differences occurred among the physicians (p<0.05). Both of studies did not show overall consistency in the preference of the medicine in the location and the specialty, except the healthcare institutions. Our findings suggest that the respondents had low preferences for generic drugs which could have resulted in limited use of generic drugs in Korea.

Opinion on the generic drugs prescription, almost all of the respondents (2011:87.5%; 2013:86.5%) stated when they prescribe generic drugs, they significantly considered generic drugs are therapeutically equivalent and just as safe as original drugs. Minor factors were the pharmaceutical company’s reliability, price, pharmaceutical companies’ marketing activity, hospital policy and others (Tables 2 and 3). To promote generic drugs prescriptions, PMS for quality maintenance (32.6%), BE test validity (26.9%), and BE results reliability (24%) were seen as the three most important factors, followed by price reduction (10.9%) and incentive policies for prescribing generic drugs (5%). Regarding willingness to prescribe generic drugs, if generic drugs quality was improved by a transparent, strong and efficient regulatory system such as the mandatory systems of audit trail and GMP, DMF enlargement and PMS, 83% stated that they would be willing to prescribe generic drugs more. The 2011 results showed that if generic drugs’ quality was guaranteed by several programs (as mentioned above), almost all of respondents would be willing to increase future generic drugs prescriptions. Therefore, the Ministry of Health and Welfare (MoHW) and MFDS were needed to establish related policies and systems to improve the quality of generic drugs. In fact, since 2012, the MFDS took the step of demanding that all companies, with generic drugs either already on the market or in the marketing approval process, re-submit their registration details, including detailed BE reports, stating in which country and laboratory the tests had been carried out [26]. In line with new drug pricing plans, the MFDS will make a promotional video and launch the campaign for the public [27]. Also, the Korean government introduced a new pricing system where drugs composed of the same ingredients should have the same price starting in January 2012. It was essential that a new pricing policy should gain the trust of the BE results related generic drugs approval.

The 2013 survey was designed to investigate physicians’ attitudes toward generic drugs and their prescribing behaviors after the drug pricing policy reform. After the introduction of the new drug pricing system, there will eventually be no difference in the price between original drugs and the generic drugs. So it would be concerned that the prescribing pattern changed more frequently from generic drugs to original drugs. But based on our results, more than a third of the respondents (35.5%) did not change their prescription pattern and a third of the respondents (29.7%) switched from original drugs to generic drugs. Our results showed that physicians’ perceptions about generic drugs prescription were little changed after the introduction of the new drug policy.

Our study didn’t include prescription pattern monitoring data, therefore, there could be a gap between the physicians’ responses and their actual prescribing behavior. Thus, future monitoring and further studies in prescribing behavior are needed. The Korean NHIS is operated by the single national insurance payer and for the country’s entire population [28]. The rapid growth of pharmaceutical expenditure has threatened the sustainability of the NHIS in Korea. Accordingly, the Korean government introduced several pharmaceutical policies to ensure rational, efficient and cost-effective supply and use of drugs as well as to limit increases in pharmaceutical expenditures. In Korea, a physician prescribes a drug, and a pharmacist dispenses the drug [29]. Therefore, understanding physicians’ attitudes and opinions towards the policy could be a key factor to promote and develop effective and efficient policies.

The last question was the physicians’ opinion on cost effective and rational use of drugs. Based on our results, in order to improve the use of cost-effective drugs, it was necessary to introduce an incentive policy, improve confidence in generic drugs and provide accurate information about the medicine. Gaining trust in generic drugs BE results is essential to the success of the new drug pricing policy. The MoHW and MFDS will try to raise the general trust in generic drugs quality. However, this will require consistent, long-term.

This research aimed to evaluate the physicians’ knowledge of generic drugs, their preferences, the reasons for using original drugs, the factors affecting willingness to use generic substitutions and their opinions on the drug policy. We achieved a good response rate, which was comparable with other surveys in this field, but our respondents didn’t fully represent the total physician population; The majority of the respondents were 40 to 50 years (2011:88.9%; 2013:84.2%) of age and were males (2011:91.1%; 2013:92.9%). In addition, when interpreting the results, it was necessary to be careful that they tended to favor government policies, as the participants were part-time members of the HIRA and may be biased.

## Conclusions

All of generic drugs in Korea have passed BE tests and were regarded by the MFDS as equivalent substitutes for original drugs, but the majority of the physicians who participated in this survey indicated negative perception and low preference for generic drugs. The respondents had negative perception of generic drugs, which could have resulted in limited use of generic drugs in Korea. In order to encourage the use of generic drugs and restore confidence in the quality and safety of generic drugs, it is necessary to provide accurate information about generic drugs regulatory authorized approval systems, especially with regard to BE test and results. In addition, it is important to strengthen promotional campaigns about generic drugs polices.

## Abbreviations

BE: bioequivalent
DMF: drug manufacturing form
GMP: good manufacturing practices
HIRA: Health Insurance Review Agency
MFDS: Ministry of Food and Drug Safety
MoHW: Ministry of Health and Welfare
OECD: Organization for Economic Cooperation and Development
OOP: out-of-pocket
PMS: post marketing surveillance
